# Advances in Quantitative Hepcidin Measurements by Time-of-Flight Mass Spectrometry

**DOI:** 10.1371/journal.pone.0002706

**Published:** 2008-07-16

**Authors:** Dorine W. Swinkels, Domenico Girelli, Coby Laarakkers, Joyce Kroot, Natascia Campostrini, Erwin H. J. M. Kemna, Harold Tjalsma

**Affiliations:** 1 Department of Clinical Chemistry, Radboud University Nijmegen Medical Centre, Nijmegen, The Netherlands; 2 Department of Clinical and Experimental Medicine, Section of Internal Medicine, University of Verona, Verona, Italy; Universidad Europea de Madrid, Spain

## Abstract

Assays for the detection of the iron regulatory hormone hepcidin in plasma or urine have not yet been widely available, whereas quantitative comparisons between hepcidin levels in these different matrices were thus far even impossible due to technical restrictions. To circumvent these limitations, we here describe several advances in time-of flight mass spectrometry (TOF MS), the most important of which concerned spiking of a synthetic hepcidin analogue as internal standard into serum and urine samples. This serves both as a control for experimental variation, such as recovery and matrix-dependent ionization and ion suppression, and at the same time allows value assignment to the measured hepcidin peak intensities. The assay improvements were clinically evaluated using samples from various patients groups and its relevance was further underscored by the significant correlation of serum hepcidin levels with serum iron indices in healthy individuals. Most importantly, this approach allowed kinetic studies as illustrated by the paired analyses of serum and urine samples, showing that more than 97% of the freely filtered serum hepcidin can be reabsorbed in the kidney. Thus, the here reported advances in TOF MS-based hepcidin measurements represent critical steps in the accurate quantification of hepcidin in various body fluids and pave the way for clinical studies on the kinetic behavior of hepcidin in both healthy and diseased states.

## Introduction

Protein profiling by time-of flight mass spectrometry (TOF MS) is based on polypeptide enrichment by selective binding to a (bio)chemical surface prior to TOF MS of retained proteins and peptides [1]. This technique is widely used to address several biomedical questions in the proteomics field, e.g. for the discovery of disease related biomarkers in biological fluids [2], [3] for protein interaction studies [4] and for immunoproteomics-based approaches [5]–[7]. Furthermore, TOF MS-based assays have the potential to simultaneously distinguish and quantify multiple isoforms/variants of a particular protein/peptide in contrast to most ELISA-based assays [5], [8]–[11]. In all these application areas, reliable quantification is imperative. Clinical mass spectrometry assays optimally use internal standards to correct for recovery, variable ionization and suppression of the molecule under analysis [12], [13].

Hepcidin is a 25-amino acid peptide that is synthesized in hepatocytes and secreted in the plasma. It binds to the cellular iron export channel ferroportin and causes its internalization and degradation [14] thereby decreasing iron efflux from enterocytes and macrophages into plasma reviewed in ref [15]–[17]. Increased iron stores and inflammation induce hepcidin synthesis, whereas suppression occurs during hypoxia and increased and/or ineffective erythropoiesis. Furthermore, hepcidin deficiency plays a central role in the iron loading in hereditary hemochromatosis and thalassemia's.

Notwithstanding recent progress, much work remains in defining the role of hepcidin in both healthy and diseased states. However, to date, few investigative tools are available [15]–[17]. The development of immunochemical methods based on the production of specific hepcidin antibodies is difficult due to the small size of hepcidin (25 amino acids; hepcidin-25 [hepc25]), and its conservation among animal species, complicating the elicitation of an immune response in host species. To date, mainly the antibody-based dotblot assay described by Nemeth et al. has successfully been used to (semi) quantify hepcidin in urine [18]–[21]. However, due to its quite laborious procedure, and its unsuitability for serum, this assay is not optimal for measurements in large clinical studies. By means of surface enhanced laser desorption/ionisation (SELDI-)TOF MS technology, we and others were successful to semi-quantify hepcidin and its isoforms in urine and serum [9], [10], [22], [23]. Very recently, other serum hepcidin assays were reported that exploited liquid chromatography tandem mass spectrometry (LC-MS/MS [24], [25]). After protein precipitation and peptide extraction, a mixture of serum and internal standard could be analyzed by LC-MS/MS. The use of non-hepcidin related peptides as internal standard, however, may affect the accuracy and reproducibility of the hepcidin concentration levels.

Here, we describe an update of the TOF MS hepcidin method for both serum and urine with considerable improvements on sensitivity, reproducibility, value assignment and quantitative abilities. This facilitates the exchangeability of studies performed by the few other available methods to date and provides the long sought tool to study hepcidin kinetics.

## Results

To overcome several of the technical limitations that previously interfered with the robustness of hepcidin measurements by TOF MS, we have systematically reconsidered all analytical steps and made improvements wherever possible. This experimental survey and the subsequent evaluation of the improvements are described below, whereas technical details can be found in the Material & Methods section.

### Recovery

Relatively large sample volumes (500 µL) obtained by dilution of the samples prior to sample preparation resulted in higher hepcidin peaks compared to our previously reported procedure in which samples were in a much smaller (5 µL) final volume [10]. This increased recovery of hepcidin is likely ascribed to decreased sticking of hepcidin with the increase of the (sample volume)/(tube surface) ratio or to less aggregation with the decrease of the hepcidin concentration. This updated procedure improved considerably the sensitivity of the method for the detection of hepc25 (see below).

### Oxidation

**Figure 1:**
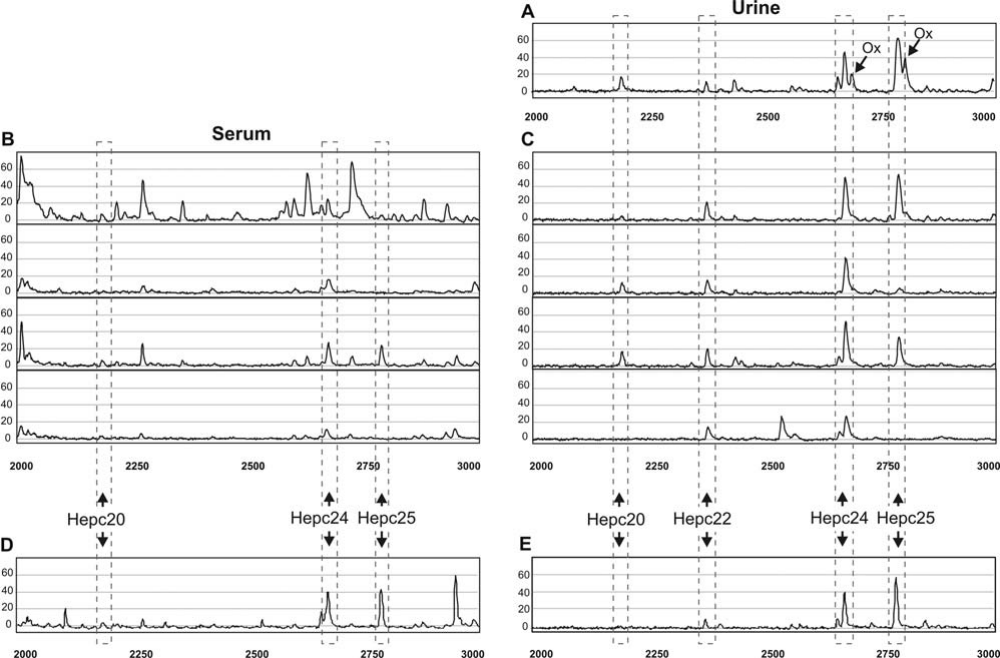
SELDI-TOF MS profiles obtained in the different experiments. SELDI-TOF MS profiles of (A) hepidin-24 spiked urine sample showing next to the expected hepcidin forms also methionine oxidized (Ox) forms of Hepc24 and Hepc25; (B and C) different patient sera and urines, respectively, spiked with Hepc24 (5 nM into urines and 10 nM into sera). Note, the influence of the serum and urine matrices on the peak height of the Hepc24 spiked to patient samples; (D and E), blank serum and urine samples spiked with both Hepc24 and Hepc25 (7.5 nM of both hepcidin forms into urine and 10 nM into serum). Note that the method appears to be more sensitive for Hepc25 than for Hepc24, with an average peak intensity ratio Hepc24/Hepc25 of 0.693. This is probably due to the absence of a negatively charged aspartic acid residue in Hepc24, which negatively affects its binding on the IMAC-Cu^2+^ protein chip surface. The hepcidin isoforms Hepc20, Hepc22 (only in urine), Hepc24 (synthetic analogue) and Hepc25 are indicated by arrows.

In some urinary samples the methionine residue of hepc24 (2673.9 Da) and hepc25 (2789.4 Da) were prone to oxidation (to [D]THFPICIFCCGCCHRSKCGM-OxCCKT, 2689.9 Da and 2805.4 Da, respectively) at experimental conditions [26]. This ambient ozone induced oxidation artifact was minimized by carrying out sample preparation in a nitrogen atmosphere by the use of an incubator with a nitrogen inlet. Oxidation peaks of hepcidin in the MS spectra were completely absent in urine samples prepared under these conditions, whereas they were clearly visible in some spectra of samples loaded on the arrays in ambient air (Figure 1A). In our hands methionine oxidation of *serum* hepcidin was never observed.

### Standard curves

Standard curves of the internal standard hepc24 were constructed by serially diluting hepc24 (0–20 nM) in tubes with blank urine and serum to an end volume of 500 µL, immediately applied to IMAC-Cu^2+^ Chips and processed according to protocol and measured by MS. Linear standard curves were obtained for hepc24 in blank urine (y = 8.62x−0.49; R^2^ = 0.996) and blank serum (y = 4.90x+3.97; R^2^ = 0.994).

### Matrix influences

To detect possible matrix influences on the flying behavior in the mass spectrometer, hepc24 was added to diverse urine and serum samples from our collection that have been shown to contain various concentrations of endogenous hepc25 [10]. We found that when spiked with the same hepc24 concentration, hepc24 peak intensities were lower for serum compared to urine. This indicates that the protein-rich serum matrix suppresses the hepcidin signal, possibly by a high competition for binding sites on the chip surface. Furthermore, we observed that the recovery of hepc24 also differs between individual serum or urine samples (Figure 1B and C), which may be attributed to distinct matrix-dependent hepcidin ionization efficiencies.

### Hepc24/hepc25 ratio

As we aimed to base our quantitative hepc25 concentration measurement on the level of the hepc25 peak intensity relative to that of hepc24, we set out to determine the hepc24/hepc25 intensity ratio's in blank urine and serum samples, that were all spiked with both compounds in duplicates of 5 different concentration combinations (Figure 1D and E). Results revealed no influence of the internal standard hepc24 to the peak height and position of the human hepc25, making a competition between hepcidin 24 and 25 for binding to the chip and ionization unlikely. Notably, the IMAC-Cu^2+^ method appears to be more sensitive for the hepc25 than for the hepc24 analogue, with mean (SD) peak ratio's hepc24/hepc25 of 0.709 (0.058, n = 10) for serum, and 0.678 (0.071, n = 10) for urine, with an average of 0.693 (0.066, n = 20) for both body fluids. The observation of similar ratio's for both urine and serum matrices, however, suggests that the intensity ratio is specimen independent.

### Reproducibility

Intra-chip or spot-to spot variation of hepc25 measured for the urine application ranged from 6.1% at 3.2 nM to 7.3% at 1.2 nM (n = 8). Similarly, precision was also good for the serum application with a CV of 5.7% at the higher level (4.4 nM, n = 8) and 11.7% at the lower level (1.8 nM, n = 8). Comparison of these CV's (average value of 7.7%) with a CV of around 12% obtained previously without internal standard [10] indicates that the use of an internal standard improved precision of the hepc25 assay, mainly by its ability to correct for differences in spot quality and instrumental settings.

### Lower level of detection (LLOD)

**Figure 2:**
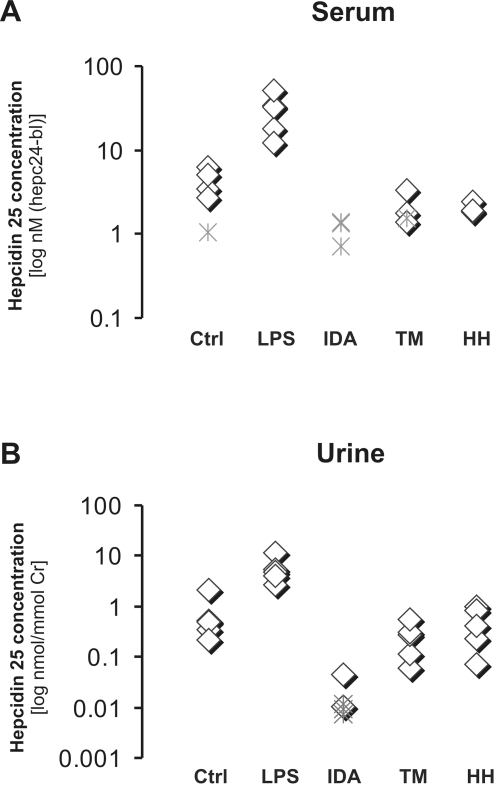
Effect of the use of an internal standard on the hepcidin-25 concentrations in urine and serum of selected clinical populations. Hepc25 concentrations were calculated in nM based on the known concentration of spiked hepc24 in serum (A) and urine (B) samples. For serum, hepc24 intensities were corrected for the background intensity of unspiked samples (hepc24-bl). Note that for both urine and serum specimens the LLOD depends on the individual sample matrix and therefore varies between samples. The LLOD was determined in the 25 human serum and urine samples by using the background intensities at m/z 2400, 2515, 2846 for serum and at m/z 2299, 2510, 2910, for urine samples, respectively. The detection limit was defined as the mean+2 SD of these measurements and found be 2.0 peak intensity for serum and 1.76 peak intensity for urine. The lower level of detection (LLOD) in nM of each individual sample was determined by incorporating the sample specific hepc24 peak intensity value and these mean LLOD values in peak int for hepc25 peak at 2789 m/z in the formulas 1 and 2 for urine and serum (see Material and Methods section). Ctrl, control; LPS, volunteers injected with polysaccharide (6 h after injection); IDA, iron deficiency anemia; TM, thalassemia major in various stages of disease; HH, C282Y homozygous hereditary hemochromatosis patients of various stages of disease; ◊, hepcidin concentration; Ж, sample specific LLOD, the hepcidin concentration of the sample is then<LLOD.

Based on the measured background noise in each MS spectrum (see Figure 2), the calculated LLOD values of the improved SELDI-TOF MS assay varied between 0.003–0.037 nM/mmol creat for urine samples. This detection limit appeared to be lower than that reported by Nemeth *et al.* [20] with a detection limit of 0.43 nM/mmol creat (10 ng/mg creat; Table 1). Remarkably, for serum samples the LLOD ranged from 0.55 and 1.55 nM, which is a significant improvement from the approximate 22 nM described for our previous procedures [10] and that of 16 nM reported by Tomosugi *et al* [22] in a similar procedure. Furthermore, it is within the range of the detection limits of 0.3–1.8 nM (1–5 ng/ml) described recently by Murphy *et al.* [24] and Murao *et al.* [25] using more laborious LC-MS/MS procedures (Table 1).

**Table 1:** Comparison of hepcidin results between recent studies.

Method		SELDI		SELDI		LC/MS-MS		Immunodot
reference		this report		Tomosugi [22]		Murphy [24]		Nemeth [18], [20], [21]
**urine**	n	nM/mmol creatˆ	n	nM/mmol creatˆ	n		n	nM/mmol creatˆ
LLOD		0.003–0.037*		n.d.		n.d	n.p.	0.43
controls	5	0.21–2.14		n.d.		n.d.	105	0.43–8.54
IDA	5	<0.011–0.045		n.d.		n.d.	±2	±0.21–0.30
inflammation	5	2.63–11.8		n.a.		n.d.	±8	±2.56–34.1
**serum**	n	nMˆ		nMˆ		nMˆ		
LLOD		0.55–1.55#		16		0.36		n.d.
controls$	5	<1.04–6.18		n.a.	10	<0.36–16.3		n.d.
IDA	5	<0.71–<1.38		n.d.		n.d		n.d.
Inflammation	5	12.1–51.4		n.a.		n.d.		n.d.

Data are presented in ranges as data from the current study do not allow calculation of means and SD's and the raw data of previous studies are only partially available.

LLOD; Lower limit of detection; n.d., not determined; n.a., not applicable; n.p., not provided.

* exact value depend on the individual urine and the creatinine concentration.

# exact value depend on the individual serum.

ˆ conversion factors: nM to µg is 2.789; from nM/mmol creat to ng/mg creat is 23.44.

$ in a rigorously defined group of healthy controls (see Table 2) these figures are: n = 23, range 0.86–12.43 nM.

### Clinical evaluation in patient groups

After clinical diagnosis we categorized urine and serum sample pairs from previous studies [9], [10], [19], [27] into 5 clinical groups of 5 patient each and quantified the hepc25 levels after spiking of hepc24 as internal standard. Urine and serum hepcidin values of the individual patients correlated significantly (ρ = 0.783, *p* = 0.002). Figure 2 illustrates the urine and serum hepc25 levels for the different categories of patients. In fact, the urine and serum hepcidin values in the (small) patient groups increased progressively from IDA to inflammation, with intermediate values in controls that differ from both patients with IDA (serum p = 0.10, urine p = 0.009) and inflammation (serum p = 0.009, urine p = 0.009) (Table 1 and Figure 2). Note that the hepcidin values of thalassemia and hemochromatosis patients are not particular informative because of individual differences in extent of anemia and treatment. The hepcidin concentration ranges we found for the patient groups are comparable to the scarce data reported by a few other groups (Table 1 [18], [20]–[22], [24]. Nevertheless, international inter-laboratory comparisons of accuracy and precision of the hepcidin assays in well defined and larger samples of patients with the various methods available to date, are warranted to increase insight and exchangeability of the hepcidin results between studies (analogous as performed previously for non-transferrin-bound-iron [28]).

### Correlation with serum iron indices in healthy control subjects

**Figure 3:**
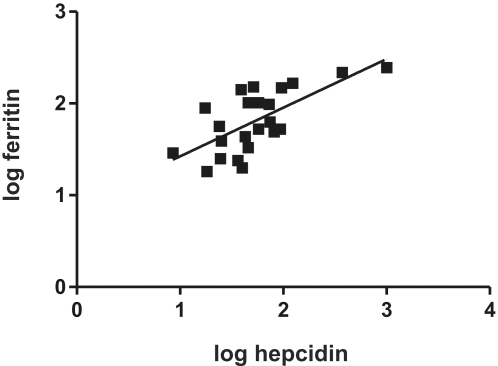
Correlation between serum hepcidin and ferritin. Serum hepcidin levels in 23 healthy volunteers (Table 2), as determined by our updated MS method, were correlated with their ferritin levels. Values were log transformed prior to correlation analysis. Pearson correlation: 0.6804 (p = 0.0004).

We evaluated the serum assay by correlating the serum hepcidin levels with various serum iron indices in 23 volunteers. Sample characteristics are presented in Table 2. Hb, serum ferritin, and body iron levels were significantly higher for the males than for the females, whereas serum iron, transferrin saturation (TS), soluble transferrin receptor (sTfR) and hepcidin levels were similar for both sexes. Within the strict selection criteria adopted, serum ferritin levels in these subjects are anticipated to represent a reliable measure of body iron [29], especially when considering a formula that includes the levels of serum transferrin receptor [30]. Indeed, serum hepcidin levels correlated significantly with both ferritin and estimated body iron stores in the total group and in men (Figure 3; Table 3). For women this was only significant for body iron stores. These findings are in agreement with previous findings on the increase of human hepcidin synthesis by iron stores [18], [22], [31], [32] and thereby demonstrate the usefulness of the hepcidin assay in clinical studies.

**Table 2:** Characteristics of healthy control subjects.

	All	Males	Females	P
n	23	12	11	
Age (years)	28 (25–31)	30 (26–35)	26 (22–30)	0.093
Hb (g/dl)	14.1 (13.6–14.6)	14.9 (14.5–15.3)	13.2 (12.6–13.8)	<0.0001
Iron (mmol/l)	17.6 (15.2–20.0)	17.1 (14.9–19.3)	18.2 (13.1–23.2)	0.656
Ferritin mg/l)	64.6 (46.1–90.6)	111 (75.9–164)	35.7 (26.5–48.1)	<0.0001
TS%	27 (23–31)	27 (23–32)	27 (20–33)	0.844
sTfR (mg/l)	1.1 (0.9–1.2)	1.1 (0.9–1.2)	1.0 (0.9–1.2)	0.622
Body iron (mg/kg)	0.3 (−2.6 to 3.2)	4.72 (1.25 to 8.20)	−4.43 (−7.27 to −1.59)	<0.0001
Serum hepcidin (nmol/l)	5.3 (3.5–8.3)	7.2 (3.3–16.0)	3.9 (2.7–5.4)	0.139

Data are means with 95% C.I. and p-values of males *vs* females by t-test. TS, transferrin saturation; sTfR, soluble transferrin receptor.

Body iron stores were calculated on the basis of the logarithm of the concentrations in micrograms of serum transferrin receptor/serum ferritin (TfR/ferritin ratio) and expressed as milligram per kilogram body weight, as follows: body iron (mg/kg) = −[log(TfR/Ferritin ratio) −2.8229]/0.1207 [30]. Positive values represent the iron surplus in stores, while negative values represent iron deficit in tissues.

**Table 3:** Correlation of serum hepcidin levels with iron indices in controls with rigorously defined normal iron status.

Iron indices	All	Males	Females
Hb	0.08	−0.44	0.01
iron	0.13	0.13	0.37
TS (%)	0.40	0.52	0.42
ferritin	0.68**	0.76**	0.57
sTfR	−0.25	−0.27	−0.45
Body iron	0.72**	0.78**	0.65*

Data are correlation coefficients by Pearson correlation. STfR, soluble transferrin receptor; TS, transferrin saturation. Hepcidin and ferritin values were log transformed prior to correlation analysis ^*^:P<0.05; ^**^: P<0.01.

### Fractional excretion of hepcidin

As shown in Table 1, value assignment in the current study shows for the first time that the urine and serum hepcidin levels appear to be of a similar magnitude. This suggests that after filtration, hepcidin is almost completely reabsorbed by the proximal tubulus, similarly to other small proteins, such as β2-microglobulin. In fact, estimation of the fractional excretions of hepcidin from urine-serum control pair samples resulted in a values between 0–3%, pointing towards a 97% to a nearly 100% tubular reabsorption of the freely filtered serum hepcidin (Table 4).

**Table 4:** Fractional excretion of hepcidin.

Disease	n	fractional excretion (%) sample		
		1	2	3
Ctrl	3	1.0	2.3	0.6
LPS	1	2.0	n.a.	n.a
TM	3	0.1	1.0	1.2
HH	3	2.8	2.9	1.6

The fractional excretion (FE) is calculated by: urine hepcidin (nM)×serum creat (µM)×100/serum hepcidin (nM)×urine creat (mM)×1000. n, the number of pairs for which all data needed to calculate the FE were available. Ctrl, controls; LPS, volunteers injected with polysaccharide (6 h after injection); TM, thalassemia major in various stages of disease; HH, C282Y homozygous hereditary hemochromatosis patients at various stages of disease; tubular reabsorbtion (%) = 100- FE (%).

## Discussion

In this study we have described an updated TOF MS method for both serum and urine hepcidin with considerable improvements on sensitivity, reproducibility, value assignment and, importantly, quantitative abilities which are critical to allow the exchangeability of studies performed by the few other available methods and to study hepcidin kinetics. Our data clearly demonstrate the added value of hepc24 as an internal standard for mass spectrometry as it, once spiked to a sample, controls for the variation in hepc25 outcome due to differences i) in peptide recovery during sample preparation, ii) changes in ProteinChip quality, iii) variable ionization and ion suppression, and iv) changes in instrumental performance of the mass spectrometer (this study; our unpublished observations).

A notable finding was the fact that hepc24 and hepc25 showed different binding characteristics when IMAC-Cu^2+^ chips were used as selective surface for protein binding. Possibly, binding of hepc24 is impaired because it lacks the amino-terminal aspargine residue of hepc25 (Material & Methods), which may affect metal affinity of hepcidin [33]. In this study, however, we decided to continue measurements with IMAC-Cu^2+^ chips to allow comparison with previous work [10] by multiplying the concentration obtained by equation 1 and 2 (Material & Methods) with the hepc24/hepc25 ratio as a constant. Importantly, our recent analyses showed that cation exchange-based enrichment of hepcidin yielded a hepc24/hepc25 ratio of 1 (data not shown), which implies that both peptides have similar binding characteristics at this surface. This clearly underscores that the specific recovery of hepcidin is dependent on the employed chromatographic chemistry for protein binding. Therefore, we strongly recommend controlling for the hepc24/hepc25 ratio on a regular basis by incorporating hepc24/hepc25 spiked blanks in future (updated) measurement protocols.

The most valuable feature of the updated hepcidin assay is the fact that the combination of selective protein binding with sensitive mass detection is uniquely capable of accurate quantification of hepcidin in *both* serum and urine (see Table 1). Importantly, inclusion of the internal hepc24 standard corrects for urine/blood matrix-differences, which up till now strongly affected the measured hepcidin peak intensities. This allowed for the first time the calculation of the fractional excretion of hepcidin, which appeared to be <3% in the analyzed serum/urine pairs. Thus, these advances have paved the way for large (pre)-clinical studies to investigate tubular reabsorption of hepcidin in various clinical disorders, especially in anemias that are associated with renal diseases where hepcidin is predicted to be an important contributor [34].

From a clinical standpoint, the availability of an accurate *quantitative* evaluation of *serum* hepcidin represents a substantial progress. Previous reports casted doubts on the reliability of determining hepcidin in serum, since it did not correlate well with either clinical diagnosis or laboratory markers of iron metabolism [35]. Several explanations for these unexpected discrepancies were postulated, including a possible too rapid clearance of this small peptide from circulation, suggesting *urinary* hepcidin (normalized to glomerular filtration) as a better estimate of hepcidin production than single-point assay in serum. The data obtained with the present updated quantitative method, provide evidence for both good correlation with iron indices in appropriate controls (Table 3), and proper detection of peptide variations for diagnostic purposes (Figure 2). This indicates that we may have now reached a sufficient level of analytic sensitivity to make that the serum hepcidin measurements can now be used as a tool in clinical studies

Altogether, this study shows the potential of high-throughput TOF MS-based diagnostic assays, especially in the case of small peptides that are difficult to handle in ELISA-based immunological approaches. Importantly, MS can provide additional information on post translational modifications, as exemplified by the discrimination between the hepc20, -22, and/or -25 isoforms in serum and urine. “However, it goes without saying that several scientific questions have to be addressed and further assay improvements should be established before hepcidin measurements can be implemented in general clinical laboratories. These issues comprise, but are not limited to, standardization, mass resolution, healthy reference values, correspondence serum and urine hepcidin, influence of potential hepcidin carrier proteins, cross-assay validation, which are all subject of our ongoing investigations. Nevertheless, we anticipate that the here described and further advances in TOF MS-based approaches can be instantaneously implemented in dedicated laboratories to quantify hepcidin levels in a broad range of biological specimens and thereby will be valuable to further unravel the role of this peptide hormone in health and disease. In this respect, it may be appreciated that future updates of our quantitative TOF MS hepcidin assay will be posted on the website “www.hepcidinanalysis.com”.

## Materials and Methods

### Patient samples

Study participants consisted of subjects that were randomly selected from our sample collection described previously [10], and additional volunteers with normal iron parameters. Participants of our previous study included 25 urine-serum sample pairs from 5 healthy volunteers (laboratory personnel and some of their spouses), 5 hereditary hemochromatosis (HFE C282Y-homozygous) patients (various stage of phlebotomy), 5 iron deficiency anemia patients, and 5 thalassemia patients treated with chelation therapy. The patients were recruited by their physicians during outpatient clinic visits (all in the Radboud University Nijmegen Medical Centre, Nijmegen, the Netherlands, except for the thalassemia major patients, who were in Ospedale Sant'Eugenio, Rome, Italy). Endotoxemia samples (n = 5) from volunteers were obtained as previously described [19]. Hepcidin blank sera and urines were obtained from a patient with juvenile hemochromatosis due to a novel hemojuvelin mutation that was shown to have hepcidin levels below the detection limit of the method [36].

Twenty-three additional controls (12 men and 11 women) with strictly normal iron status were enrolled in Verona as previously described [23]. Briefly, they were selected among healthy volunteers participating in a phase II trial at the Centre for Clinical Research of the Azienda Ospedaliero-Universitaria di Verona, Italy. At enrollment, they completed a questionnaire with specific items relevant to iron metabolism (i.e. any history of blood donations, previous pregnancy, menstrual losses, etc.) and were evaluated by laboratory studies including complete blood count (CBC), serum iron, transferrin saturation, ferritin, C-Reactive Protein, soluble Transferrin Receptor (sTfR), liver function tests, and creatinine. To be considered as appropriate “normal controls” for the hepcidin assay by SELDI-TOF-MS, all these parameters were required to be normal.

Written informed consent was obtained from all study participants, according to the Declaration of Helsinki. Samples were collected between December 2005 and June 2007 and stored at −80°C in aliquots to avoid multiple freeze-thaw cycles. Hepcidin assays for the current study have been performed between January and July 2007.

### SELDI-TOF MS measurements

Hepcidin measurements by SELDI-TOF-MS were performed as previously described [10] and updated with i) the use of an internal standard to allow quantification and value assignments to urine and serum hepcidin levels, and ii) exploiting a working volume of 500 µL instead of 20 µL to increase recovery of hepcidin during sample preparation. In brief, after dissolving the lyophilized peptide hepcidin 24 (hepc24) in distilled water (0.5 µM), 5 µL or 10 µL of the solution was added as an internal standard to 495 µL urine (5 nM) or 490 µL serum sample (10 nM), respectively. The feasibility of this procedure was illustrated by application of a 5 µl-sample to copper-loaded immobilized metal-affinity capture ProteinChip arrays (IMAC30-Cu^2+^) that binds hepcidin based on its affinity for Cu^2+^ ions. However, also weak cation exchange bead-based approaches were successfully employed to capture hepcidin (iso)forms based on their isoelectric point >8 to allow hepcidin measurements by matrix assisted (MA)LDI TOF MS [10], [37], (our unpublished observations) . All binding surfaces were equilibrated and washed with appropriate buffers according to the manufactures instructions (Bio-Rad, Hercules, CA). Subsequent work up, SELDI-TOF MS instrumental settings, read out and data analysis are described elsewhere [10], with the addition that protein chip handling was performed in a nitrogen atmosphere to prevent methionine oxidation [26]. In previous studies, proteomic techniques showed that hepc25, hepc22 and hepc20 were present in urine, whereas only hepc25 and hepc20 were present in serum [10]. Samples with hepc25 peak heights >55 Int were considered to be out of the linear range and were diluted with blank serum or urine from a patient with juvenile hemochromatosis [36].

### Internal standard

We choose to include an internal standard in our mass spectrometry method as this enables to: i) increase the precision, ii) improve the accuracy by reducing matrix influences and to control for instrumental settings and, iii) assign a value to hepcidin concentration. As internal standard we selected hepc24 for the following reasons: i) TOF MS can clearly distinguish it's molecular weight from the endogenous human hepcidin isoforms hepc25, hepc22 and hepc20, ii) it has similar chromatographic binding and flying characteristics as the natural hepcidin isoforms and iii) its m/z position in urine and serum SELDI-profiles is in a region with relatively few other peaks; e.g. in contrast to the heavy isotopes of hepc25, hepc24 has a mass that is different from that of methionine oxidized hepc25. In our experiments we used synthetic human hepc24 (THFPICIFCCGCCHRSKCGMCCKT, 2673.9 Da, weight assessment by quantitative amino acid analysis in triplicate) and human hepc25 (DTHFPICIFCCGCCHRSKCGMCCKT, 2789.4 Da) that were obtained from Peptides International Incorporated (Louisville, KY). Noteworthy, hepc24 differs from the most abundant endogenous hepcidin form hepc25 by the absence of the amino-terminal aspargine (D) residue, whereas the natural occurring hepc22 and hepc20 isoforms lack the amino-terminal sequences DTH and DTHFP, respectively.

This experimental set up enabled us to assign values to urine (normalized to urinary creatinine values and reported in nM/mmol creatinine [creat]) and serum hepc25 (nM), which were formerly expressed as Mint/mmol creat and Mint/L, respectively [10]. For this purpose the following equations were applied for urine (1) and serum (2):

1. [(sample 2789 m/z peak intensity)×5 nM/(hepc24 spiked sample 2673 m/z peak intensity)]/mmol creat
2. (sample 2789 m/z peak intensity)×10 nM/(hepc24 spiked sample 2673 m/z peak intensity– non spiked sample 2673 m/z peak intensity)

For the sera, but not for urines, we occasionally observed minor peak at the 2673 m/z position of hepc24. Therefore, the serum protocol was adapted in that the peak intensity of the non-spiked sample was subtracted from the hepc24 peak of the spiked samples.
